# Complexity of hospital demand during the COVID-19 pandemic in Mexico City

**DOI:** 10.1371/journal.pone.0358798

**Published:** 2026-09-30

**Authors:** Guillermo de Anda-Jáuregui, José Sifuentes-Osornio, Ofelia Angulo-Guerrero, Juan L. Díaz-De-León-Santiago, Héctor Benítez-Pérez, Luis A. Herrera, Oliva López-Arellano, Arturo Revuelta-Herrera, Ana R. Rosales-Tapia, Manuel Suárez-Lastra, David Kershenobich, Rosaura Ruiz-Gutiérrez, Enrique Hernández-Lemus

**Affiliations:** 1 Population genomics, Instituto Nacional de Medicina Genómica, Mexico City, Mexico; 2 Investigadores e Investigadoras por México, Secretaría de Ciencia, Humanidades, Tecnología e Innovación, Mexico City, Mexico; 3 Instituto Nacional de Ciencias Médicas y Nutrición Salvador Zubirán, Mexico City, Mexico; 4 Secretaría de Ciencia, Humanidades, Tecnología e Innovación, Mexico City, Mexico; 5 Secretaría de Educación, Ciencia, Tecnología e Innovación, Gobierno de la Ciudad de México, Mexico City, Mexico; 6 Dirección General de Cómputo y de Tecnologías de Información y Comunicación, Instituto de Investigaciones en Matemáticas Aplicadas y en Sistemas, Universidad Nacional Autónoma de México, Mexico City, Mexico; 7 Instituto de Investigaciones Biomédicas, Universidad Nacional Autónoma de México, Mexico City, Mexico; 8 Instituto Tecnológico de Estudios Superiores de Monterrey, Monterrey, Mexico; 9 Secretaría de Salud de la Ciudad de México, Mexico City, Mexico; 10 Instituto de Geografía, Universidad Nacional Autónoma de México, Mexico City, Mexico; 11 Secretaría de Salud, Mexico City, Mexico; National Autonomous University of Mexico Institute of Geophysics: Universidad Nacional Autonoma de Mexico Instituto de Geofisica, MEXICO

## Abstract

The COVID-19 pandemic posed unprecedented challenges to healthcare systems worldwide. In densely populated urban areas such as Mexico City, hospital strain was amplified by high case volumes and limited resources. This study aims to characterize the spatial and temporal organization of hospital demand and its relationship to patient outcomes. We conducted a retrospective analysis of COVID-19 hospitalization data in Mexico City using line-list data from the SISVER surveillance system. Spatial dynamics were examined using the weighted centroid of hospitalizations, and patient–hospital interactions were modeled as a time-resolved bipartite network. The emergence of giant components was used as a proxy for system strain and evaluated in relation to patient outcomes. Hospital demand exhibited marked spatial shifts, including a northward displacement of the hospitalization centroid over time. A small group of 17 hospitals consistently managed the majority of cases. During periods of high demand, the network underwent structural transitions characterized by the emergence of large connected components spanning diverse neighborhoods. These periods of increased system-wide connectivity were associated with higher case fatality rates, particularly among patients over 40. Hospital demand reorganizes at the system level under stress, leading to loss of spatial structure and increased strain. The emergence of large connected components may serve as an early indicator of systemic overload. These findings provide a network-based perspective on healthcare system stress and may inform strategies for resource allocation and emergency response.

## Introduction

The COVID-19 pandemic posed unprecedented challenges across all sectors of society. Beyond the personal toll, healthcare systems were pushed to their limits: major institutions exceeded capacity, medical staff were overwhelmed, and critical resources became scarce [1–3]. The scale of this disruption was quantified in a landmark comparative analysis of 32 countries, which documented more than 30 million missed hospitalizations and 4 million missed surgical procedures in 2020–2021 alone, with countries that had fewer baseline healthcare workers and hospital beds experiencing disproportionately greater disruptions [4]. The spatial and temporal patterns of these pressures revealed deep-seated structural constraints, insights that are crucial for preparing for future crises of similar magnitude [5–7]. Surge-capacity models developed early in the pandemic demonstrated that even well-resourced systems lacked adequate tools to anticipate the magnitude and duration of demand spikes, underscoring the need for dynamic, data-driven approaches to healthcare planning [8]. These challenges were especially complex in metropolitan areas, where large, heterogeneous populations amplified the scale and impact of the emergency. Mexico City exemplifies such a scenario.

Mexico City, home to the highest concentration of tertiary care hospitals in Mexico, provides healthcare services not only to its 9 million permanent residents but also to an additional 13 million people from the surrounding metropolitan area and beyond. Despite this extensive healthcare infrastructure, the COVID-19 pandemic imposed unprecedented strain on the system, challenging its capacity to meet the rapidly growing demand for medical attention.

This experience is not unique: across Latin America, a region where densely populated cities with concentrated poverty were disproportionately affected, socioeconomic inequities deepened the burden on urban healthcare networks [9,10]. Within Mexico City specifically, seroprevalence surveys estimated that nearly 20% of the population had been exposed to SARS-CoV-2 by early 2021 [10], and prior work has documented that the spatial distribution of cases strongly co-varied with social marginalization across boroughs and neighborhoods [11,12].

In this regard, the pandemic exposed the (already existent, but possibly latent) inherent complexities of the health system, which operates as a multifaceted organization with numerous interdependent components. Traditionally viewed as hierarchical and linear, health systems are now recognized as complex adaptive systems characterized by non-linear interactions, self-organization, and emergent behavior [11]. This conceptual framing has proven especially useful during the pandemic: research on healthcare systems under COVID-19 stress has shown that shocks of sufficient magnitude can exceed critical adaptation thresholds, triggering qualitative changes in system behavior analogous to phase transitions [13,14]. The sudden and unpredictable nature of the COVID-19 crisis [15] further amplified these complexities, revealing both the strengths and vulnerabilities of the healthcare network in Mexico City.

The present research systematically examines the evolving patterns of hospital demand during the pandemic, highlighting the dynamic shifts in patient behavior and hospital utilization. It was observed that, as the crisis intensified, individuals often sought care at hospitals far from their residences, resulting in an uneven and entangled distribution of demand across the city’s healthcare facilities. Similar patterns of non-proximity care-seeking behavior have been documented using network science approaches in other healthcare systems. Network science is an interdisciplinary approach to complex systems that embraces complex collective behavior by modeling the interactions using networks in which the members of the collective (termed nodes, can be humans, animals, pathogens, proteins, genes, and so on) interact with each other via links (that may represent co-existence, correlation, physical, chemical or biological interaction, etc.) giving rise to emergent phenomena [16]. Network science has been successfully applied to epidemic phenomena in the past [17–20]. Along similar lines, Kohler *et al*. modeled inpatient care as networks of clinical units and patients, revealing that topological features of the network could identify bottlenecks and dysfunctional states [21]. More recently, Zhong *et al.* analyzed patient flow networks built using electronic medical records across U.S. healthcare systems and showed that, during COVID-19, cross-regional patient flows increased and network absorptivity rose by approximately 10% over pre-pandemic baseline: a finding that illustrates how stressed systems redistribute demand in ways that are not easily predicted from local capacity alone [22]. Network-based approaches have also been proposed for planning capacity across hierarchical multi-facility systems during surges [23] and for real-time surveillance of nosocomial transmission chains [24]. While some hospitals experienced consistently high demand, others saw fluctuating levels of patient load, reflecting the adaptive and often chaotic nature of the pandemic response.

By analyzing these patterns, key factors were identified as contributors to the resilience of the health system, as well as areas where it struggled to cope with the extraordinary pressures of the pandemic. These patterns are analyzed at the level of the full system, focusing on how hospital demand reorganizes under stress and how such reorganization is associated with patient outcomes. These insights may become crucial for developing strategies to enhance the system’s preparedness and adaptability in future health emergencies.

In summary, this research introduces a network-based way to measure healthcare system stress by modeling patient-hospital interactions as a bipartite network, where the emergence of “giant components” (i.e., large, entangled subgraphs encompassing over half the network) serves as a mathematical proxy for systemic overload. By analyzing COVID-19 data from Mexico City, we demonstrate that these network transitions reflect a shift toward a disorganized state where patients travel nearly twice the “optimal” distance to seek care. Most critically, this structural strain was found to correlate with higher case fatality rates for patients over 40, suggesting that monitoring network connectivity could provide a vital early warning system for predicting clinical risk during public health emergencies.

## Methods

### Epidemiological data

For this study, data from the centralized database for COVID-19 case reporting system (SISVER) was used. SISVER (respiratory disease epidemiological surveillance system) was managed by the Mexican federal government. The dataset is a “line-list” format, with each row representing an individual case. It is important to note that all information in this database is collected exclusively from official public reporting institutions at the state or federal level.

As part of the Federal Health System, our institutions were granted access to the complete dataset, comprising 130 variables per case. For this analysis, the study focused on key variables relevant to the research objectives, including the patient’s ZIP code of residence, date of symptom onset, age, hospitalization status, and the medical unit where the patient received care.

This dataset contained detailed insights into the spatiotemporal patterns and dynamics of COVID-19 hospitalizations within the study population. Only cases meeting the following criteria were included: (i) confirmed COVID-19 cases, verified through PCR or antigen testing; (ii) cases recorded in medical units or testing sites located within Mexico City boundaries; (iii) patients with a reported residence in Mexico City; and (iv) cases collected up to epidemiological week 16 of 2022 that required hospitalization. A total of 122,933 hospitalized cases were included in the analysis.

The study was approved by the ethics and research committees of the Instituto Nacional de Medicina Genómica (approval codes CEI/1479/20 and CEI 2020/21).

### Medical unit data

Data on medical units were obtained from the Catalog of Unique Keys for Medical Units (CLUES), a federal database providing information on medical units across Mexico. Using the geolocation data provided in this catalog, the road distance between each medical unit and the centroid of each ZIP code in Mexico City was calculated.

### Evaluation of distance traveled for COVID-19 medical attention

The travel behavior of patients requiring hospitalization was obtained by calculating the (road) distance they traveled from their place of residence (approximated by the centroid of their ZIP code, using in-house road accessibility data from UNAM’s Geography Institute) to the medical unit where they received treatment. The weekly weighted average distance traveled was computed using the formula:


$$\begin{array}{c} Weighted\;Average\;Distance\;=\;\frac{\sum^{cp,h}n_{cp,h\;}d_{cp,h}}{\;} \end{array}$$

Where *n*_*cp,h*_ is the number of patients from ZIP code *cp* in hospital *h,* d_cp,h_ is the distance between the (centroid of) ZIP code *cp* and hospital *h*, and n is the total number of patients requiring hospitalization in a given week. We compared this empirical distance to an *ideal* distance scenario, in which all patients received care at the medical unit closest to their residence; in this scenario, each patient is assigned to the geographically closest hospital according to the same distance metric, without considering capacity constraints.

### Hospital territorial demand network model

To capture the complexity of hospital demand dynamics throughout the pandemic, a series of weighted bipartite networks representing the relationships between ZIP codes (residential areas) and hospitals were constructed. For each week, a network with ZIP codes *cp* and hospitals *h* as nodes, connected by edges when at least one resident of ZIP code *cp* was treated at hospital *h* was created. The weight of each edge represented the number of patients from that ZIP code treated at the hospital.

Formally, each bipartite network B can be defined as


$$\begin{array}{c} B_{t}(CP,H,\;W)\; \end{array}$$

where *CP* is the set of ZIP codes for the Mexico City Metropolitan Area, H is the set of hospitals providing COVID-19 treatment, and W is the set of patients from a given ZIP code treated at a given hospital. An edge in the network exists if W_cp,h_ > 0; that is, if at least one resident of a given ZIP code was treated in a given hospital, in the time period (week) represented by that particular network.

This analysis focused on the largest connected component (LCC) of the network. A connected component of a network is a subset of nodes that may reach each other through a path of edges in the network. With this in mind, the largest connected component of a network is the component containing the most nodes out of all the components in the network. Formally,


$$\begin{array}{c} LCC(B)\;=max_{C_{i}\in C(B)}|V(C_{i})| \end{array}$$

Where C(B) is the set of connected components. The largest connected component will therefore contain the maximum number of nodes in the network, compared to all other components.

Following this, the existence of a *giant component* can be formally defined as


$$\begin{array}{c} LCC(B)\;=\;GC(B)\;iff\;|V(LCC(B))\;\geq \;\frac{V(B)}{\text{2}}+\text{1} \end{array}$$

That is, the largest connected component of a network is considered giant if it contains more than half the total number of nodes in the network.

The emergence of such giant components over time, which indicate a phase transition in the network structure, was monitored. These transitions reflect changes in hospital demand patterns, especially during the progression of the pandemic and hospital system strain (in terms of demand and capacity saturation). All network analyses were performed using *Igraph* version 2.2 [25] for R (version 4.5).

## Results and discussion

### Hospital demand is a spatially dynamic variable that changes throughout the pandemic

The COVID-19 pandemic in Mexico City presented a complex and evolving landscape of hospital demand. Contrary to the assumption that certain areas would bear a consistent burden of cases, the spatial distribution of hospitalizations shifted notably over time, often in counterintuitive ways [26]. This dynamic movement of cases reflects the heterogeneous patterns of exposure and vulnerability across different neighborhoods of the city, as well as structural biases in social development between boroughs and neighborhoods [27,28].

During the initial stages of the pandemic, cases were heavily concentrated in certain areas, primarily those with higher population density and relatively lower socioeconomic status [29]. However, as the pandemic evolved, the geographical footprint of the virus expanded, reaching new regions drastically altering the patterns of hospital demand. This dispersion of cases is indicative of varied levels of exposure across the city, influenced by factors such as population mobility, compliance with public health measures, and local transmission dynamics [12].

To capture these shifts in hospital demand, the weighted centroid of hospitalizations over time was measured, as seen in Fig 1. This metric was useful to quantify the “center of mass” of hospital demand, weighted by the number of cases in each location. This analysis revealed a gradual but significant northward movement of the centroid as the pandemic progressed. Initially, demand was focused in the densely populated southern and eastern boroughs of Mexico City. Over time, however, this centroid shifted towards the northern regions, highlighting the spatially dynamic nature of the pandemic.

**Fig 1:**
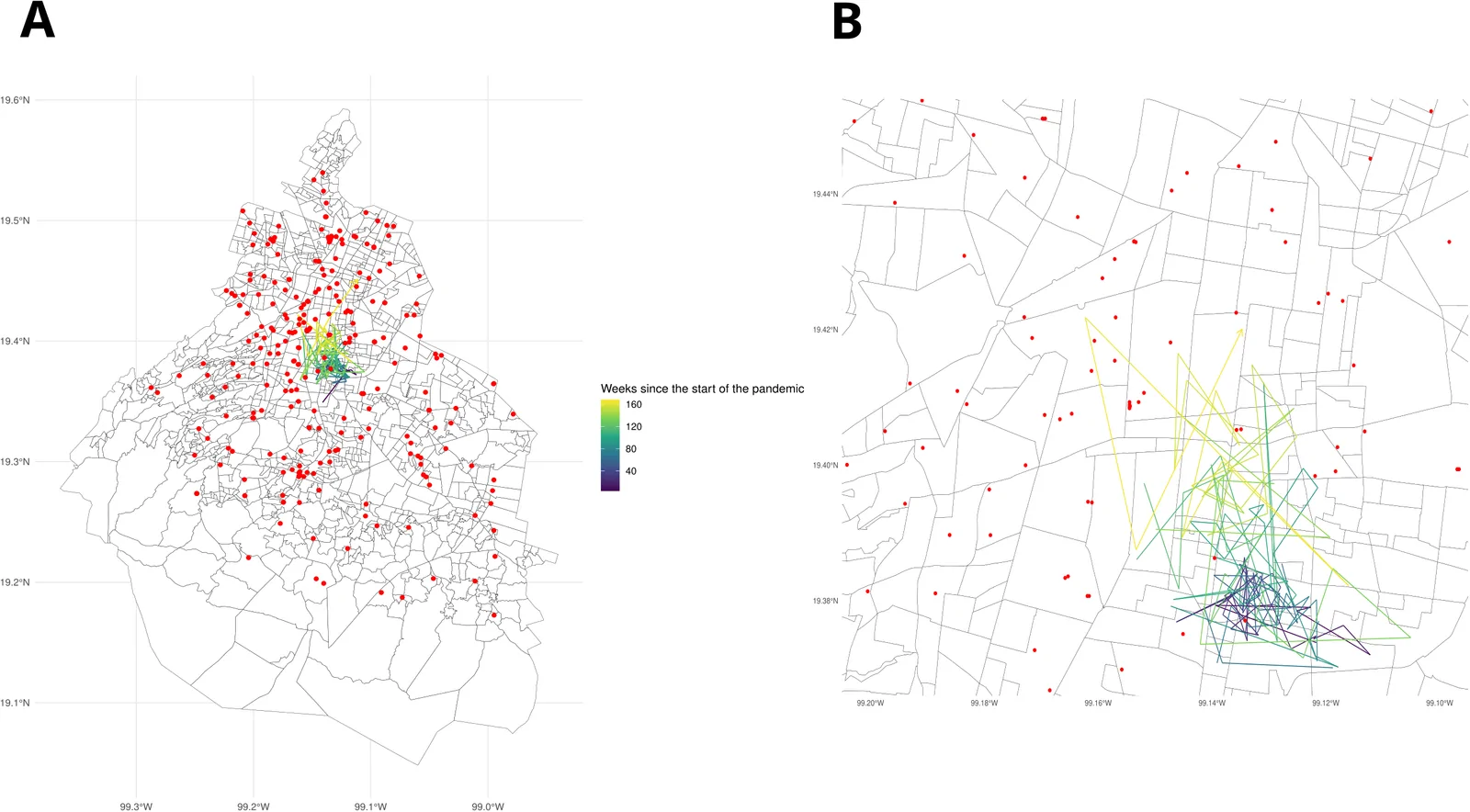
Spatial trajectory of the weighted centroid of COVID-19 hospitalizations in Mexico City over time, calculated based on patients’ place of residence (ZIP code level). The centroid represents the center of mass of hospitalization demand at each epidemiological week. Red markers indicate hospitals designated for COVID-19 care. Panel A shows the full city extent; Panel B provides a zoomed view of the centroid trajectory.

This northward drift in hospital demand underscores the adaptability of the healthcare system, which had to reallocate resources and adjust strategies in response to the changing geographical distribution of cases. Understanding these spatial dynamics is crucial for optimizing resource allocation and ensuring that all areas of the city have adequate access to medical care, particularly during periods of high demand.

Nevertheless, hospitalizations were concentrated in a few hospitals throughout the pandemic

Despite the dynamic distribution of COVID-19 cases across Mexico City, the majority of hospitalizations were concentrated in a relatively small number of medical facilities. Specifically, 17 hospitals were ranked in the top 10 in terms of admissions for at least 35 weeks, as seen in Fig 2. These hospitals, spanning various sectors of the healthcare system (including the Mexican Social Security Institute (IMSS), the Institute for Social Security and Services for State Workers (ISSSTE), the Health Secretary, and the military), handled most of the hospital demand throughout the pandemic [26].

**Fig 2:**
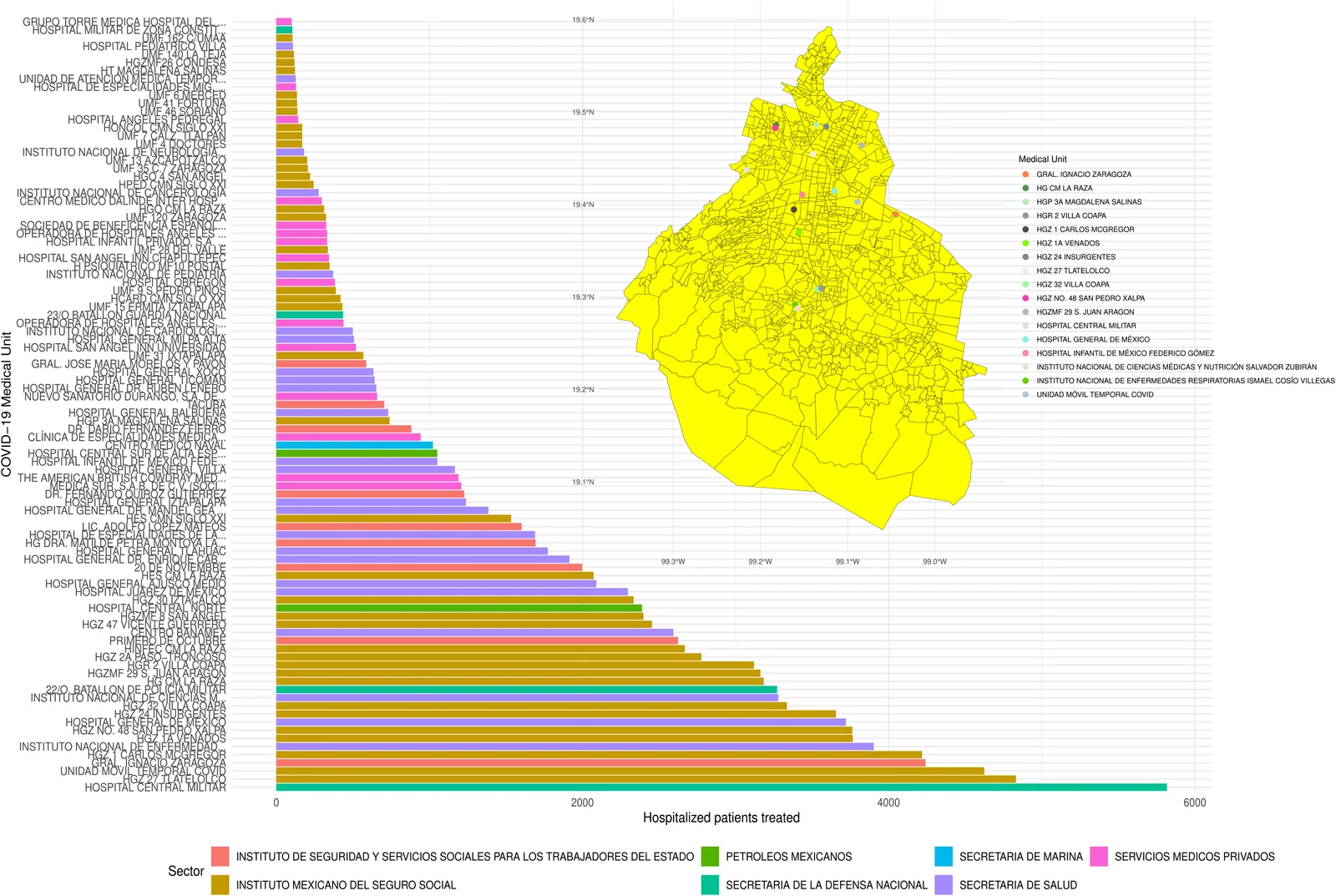
Distribution of COVID-19 hospitalizations across medical units in Mexico City. Bars represent the total number of hospitalized cases treated by each hospital over the study period. The inset map shows the geographic location of the hospitals most consistently ranked among the highest in patient volume.

The consolidation of hospitalizations in these facilities reflects a multifactorial process shaped by population distribution, healthcare system structure, and resource allocation. Understanding how these hospitals came to dominate the demand landscape during different phases of the pandemic can offer valuable insights for optimizing resource distribution in future public health emergencies. For a population as socioeconomically and geographically disparate as Mexico City, comparing the evolution of these facilities’ demand with the locations of the most affected populations highlights key patterns in access to care and resource utilization.

A distinction exists between hospitals under the Federal Health Secretary, local healthcare services of Mexico City, and those belonging to IMSS and ISSSTE. IMSS and ISSSTE hospitals regularly serve enrolled beneficiaries, while federal and local hospitals absorb a more heterogeneous patient population. Nevertheless, during the pandemic those institutions were instructed to provide services to the general population. However, there is currently no available data that quantitatively shows how this affected access to health services. Such data may provide better insights on resource allocation.

These hospitals experienced varying levels of demand over time, with most seeing significant increases during the high-contagion waves of the B.1.519 variant [30], the Delta variant, and, to a lesser extent, the first Omicron wave [31]; as seen in Fig 3. Demand waned, but did not disappear, as vaccination efforts advanced and reinfections came to dominate the epidemiological landscape [32]. During the initial phase of the pandemic, the military hospital managed a substantial portion of the hospitalizations. In subsequent waves, the special COVID-19 temporal unit, established as a dedicated response facility in a large convention center in the northwestern part of the city, absorbed the majority of hospitalizations, particularly during the B.1.519 wave. This temporary unit represented an instance of adaptability within the otherwise static hospital infrastructure.

**Fig 3:**
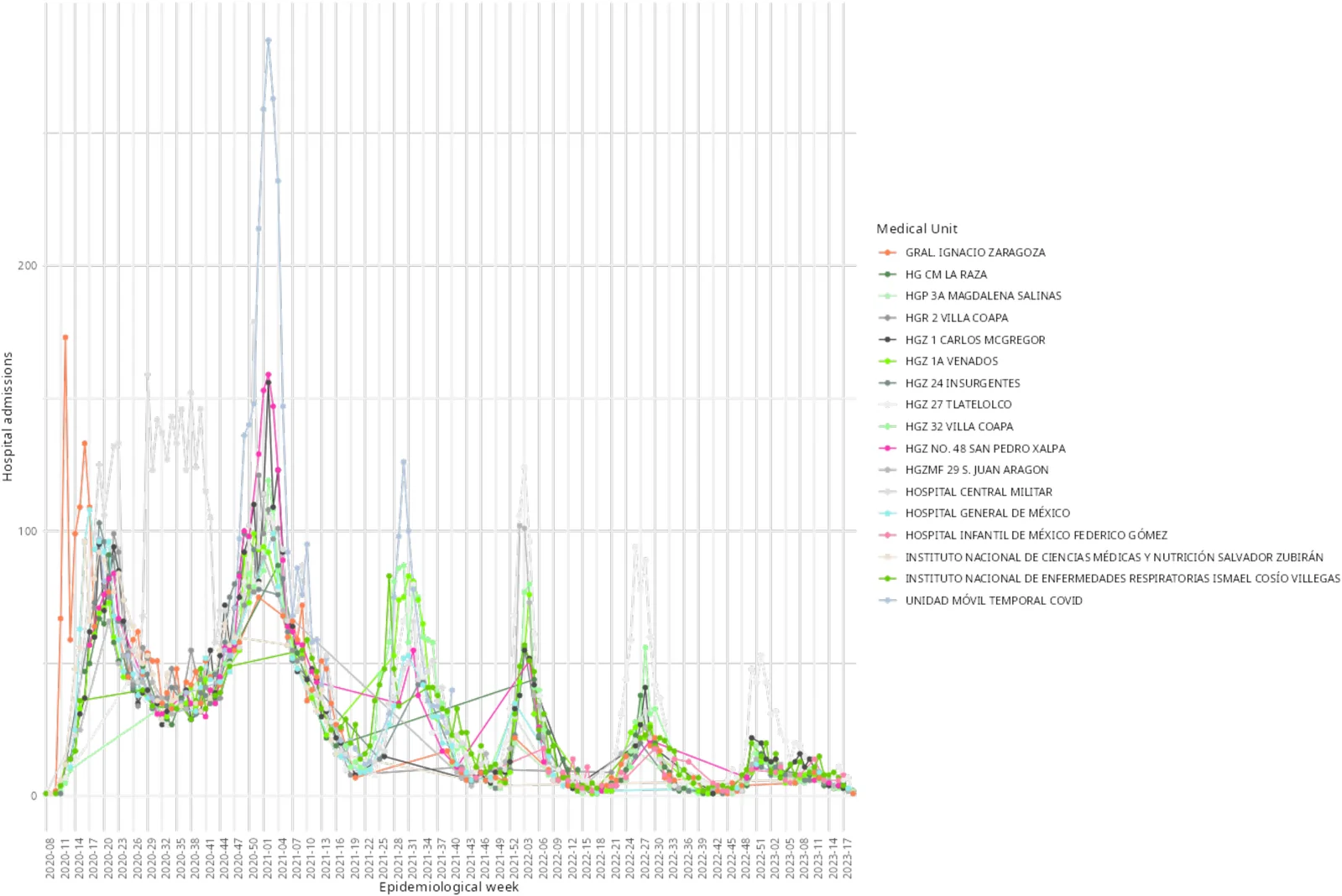
Weekly time series of COVID-19 hospitalizations in the hospitals most consistently ranked among the highest in patient volume in Mexico City. Values represent the number of new hospital admissions per week over the study period.

### Hospital attention seeking behavior did not limit itself by spatial closeness

To understand the hospital attention-seeking behavior of COVID-19 patients in Mexico City, the distance patients traveled from their place of residence to the hospital where they received care was analyzed. The analysis focused on the weekly average distance traveled, calculated from the centroid of each patient’s ZIP code to the hospital where they were treated. This distance was then compared to an “optimal” scenario, in which patients would have been treated at the hospital closest to their residence. Fig 4 highlights these results.

**Fig 4:**
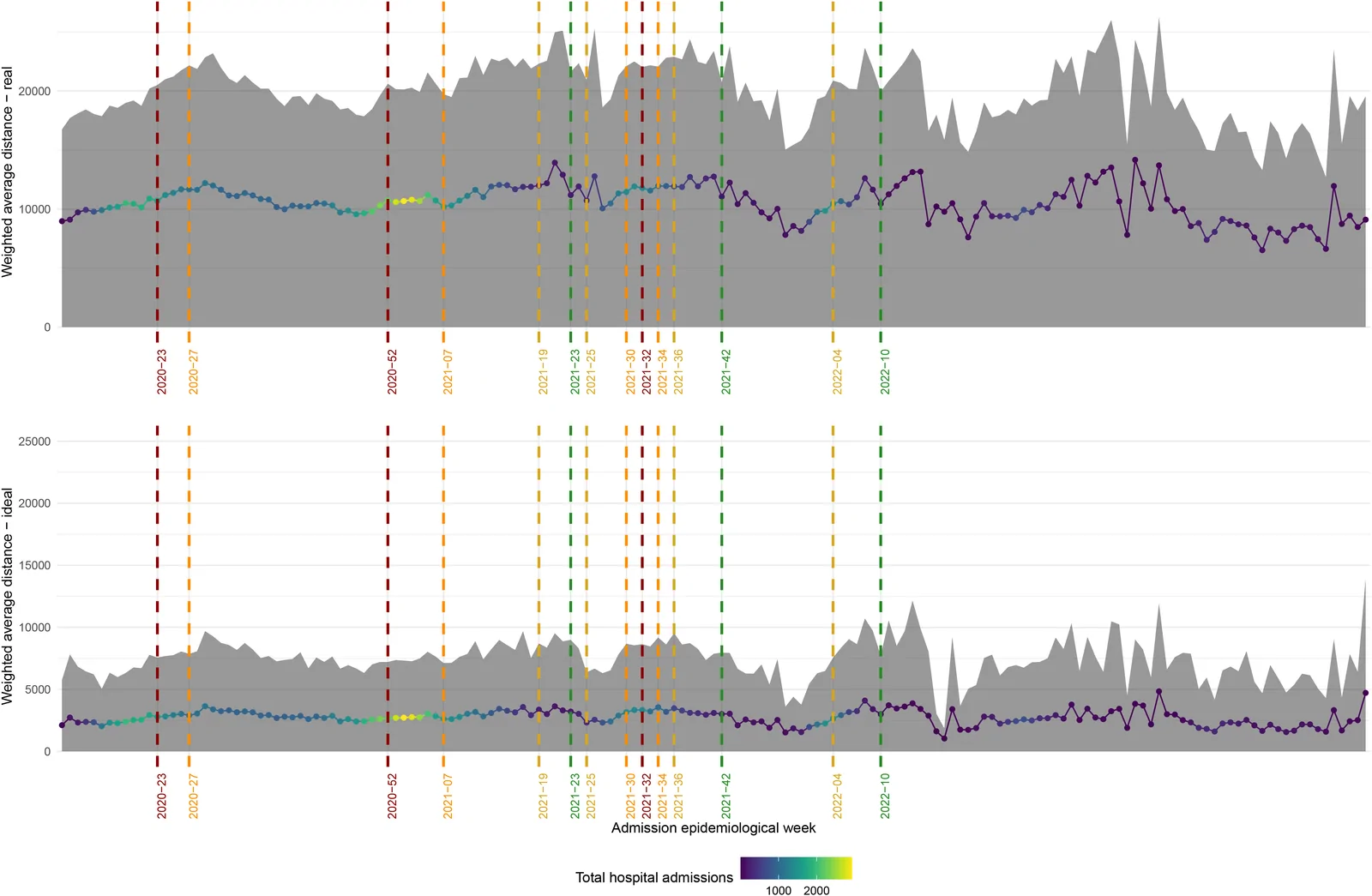
Weekly time series of the weighted average distance (km) traveled by COVID-19 hospitalized patients from their place of residence (ZIP code centroid) to the treating hospital in Mexico City. The upper panel shows empirical distances; the lower panel shows the optimal distance: assuming admission to the nearest hospital. Dotted lines indicate transitions in the epidemiological stoplight system over the study period.

The analysis revealed that the actual distance traveled by patients was nearly twice as long as the optimal distance throughout the pandemic. This discrepancy indicates that patients did not necessarily seek care at the nearest facility, even when geographical proximity would suggest otherwise. Instead, their hospital-seeking behavior was influenced by other factors, perhaps including perceived quality of care, hospital reputation, and possibly the availability of specialized services.

Moreover, the actual distance traveled by patients increased significantly during periods of high hospital saturation, such as during the peak waves of the pandemic, and following the relaxation of public health measures, such as the change in the epidemiological stoplight from red to orange. These patterns suggest that when hospital resources were strained or when restrictions were eased, patients were more likely to travel farther to seek care, possibly due to overcrowding at closer facilities or increased mobility.

The analysis of these travel patterns highlights the constraints that prevented the healthcare system from evenly distributing hospital demand, as well as the behavioral flexibility of patients in seeking medical attention. It also underscores the need for strategies to reduce these travel burdens, such as improving resource allocation and communication to ensure that patients are aware of available care options closer to home, particularly during periods of high hospital demand.

It should be noted that hospital demand fluctuated in a closely correlated way for both general hospitalization and intensive care units (ICU). This is important as it highlights the level of stress and participation of each institution facing the pandemic. In general, it is expected that a higher demand of ICU beds would be indicative of a stronger impact on resource demands. As shown in Fig 5, ICU demand scaled in considerably the same proportion throughout the first four waves. Only after vaccination efforts were widely deployed did the ICU demand decrease, as seen in the fifth wave.

**Fig 5:**
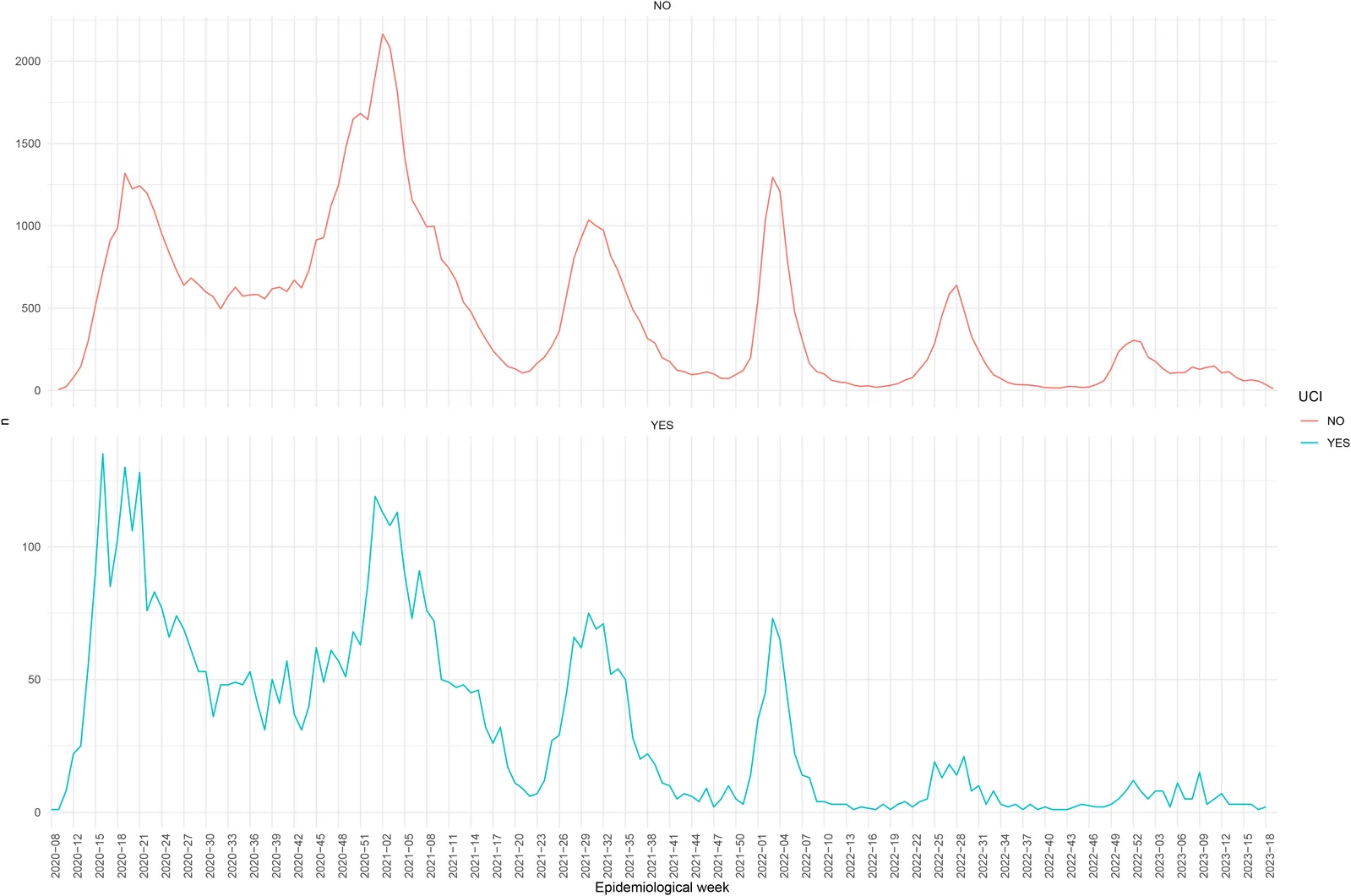
Comparison of hospital demand in Mexico City, measured as daily COVID-19 admissions to general hospitalization beds and intensive care unit (UCI, *unidad de cuidado intensivo* in spanish) beds over the study period. Values represent the number of new admissions per day in each category.

### Higher hospital demand leads to phase transition in the hospital demand network

To better understand the dynamics of hospital demand during the COVID-19 pandemic, the interaction between hospital demand and supply via a “hospital demand network” was modeled. This network is represented as a bipartite graph, where nodes are either residential areas (defined by ZIP codes) or hospitals, and connections are established when a hospital provides care to patients from a given area. The structure of this network evolves over time, as seen in Fig 6, reflecting changes in patient behavior and hospital utilization.

**Fig 6:**
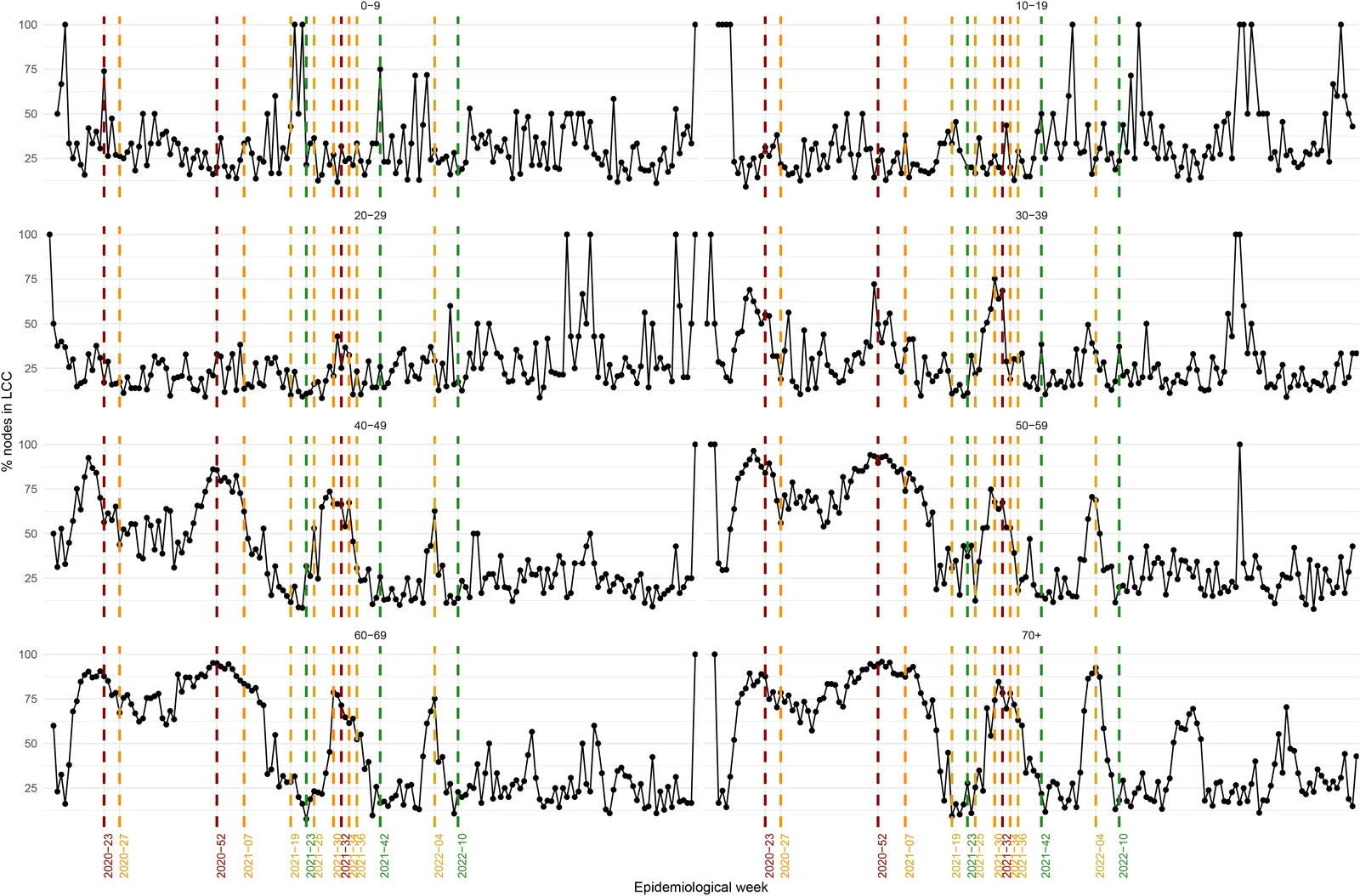
Weekly time series of the relative size of the largest connected component (LCC) in the hospital demand network, expressed as the percentage of nodes included in the LCC. Panels correspond to different age groups. Dotted lines indicate transitions in the epidemiological stoplight system over the study period.

A key feature of this network is the emergence of giant components: connected subgraphs encompassing more than 50% of the nodes in the network. These giant components are indicative of a phase transition [33,34] in the hospital demand network, a phenomenon that occurs as the system transitions from a more ordered to a more disordered state [35] in response to increased demand.

During periods of lower hospital demand, the network is more structured and ordered, with patients predominantly seeking care at hospitals located closer (although not necessarily the nearest) to their place of residence. This results in a network composed of many small, distinct components, where a small set of hospitals serve a specific set of neighborhoods. However, as demand increases, this orderly structure breaks down. Patients from various areas begin to seek care at hospitals outside their immediate vicinity, either due to overcrowding or the unavailability of care at closer facilities.

This shift leads to the formation of a giant component in the network, characterized by hospitals admitting patients from a wide range of neighborhoods. The network becomes more disordered, reflecting the strain on the healthcare system and the loss of spatial constraints on patient behavior. This phase transition is a critical indicator of system overload and occurs just before or during periods of high hospital demand, such as during peak waves of the pandemic.

The emergence of giant components in the hospital demand network highlights the nonlinear dynamics of the healthcare system under stress. Understanding these transitions can provide valuable insights into the capacity limits of the system and the points at which patient care becomes increasingly disorganized. This knowledge is crucial for designing interventions aimed at mitigating the impact of surges in hospital demand and maintaining a more stable and effective healthcare network during future public health crises.

### Higher stress in the hospital network leads to higher risk for hospitalized patients

Using the largest connected component (LCC) in the hospital demand network as a measure of “disordered strain” on the healthcare system, an increased strain, indicative of higher hospital demand, was associated with less favorable outcomes for hospitalized patients. As the LCC expands, encompassing a larger portion of the network, it reflects a breakdown in the structured distribution of patients across hospitals, suggesting that hospitals are increasingly overwhelmed and are treating patients from a broader range of neighborhoods.

This relationship between network strain and patient outcomes varied by age group; as seen in Fig 7. This association was particularly pronounced among older patients, for whom age is already established as a primary risk factor for severe COVID-19 outcomes [36]. In these older age groups, the correlation between increased network strain and adverse outcomes was not linear. Instead, it exhibited a phase transition: as the size of the giant component in the network grew, there was a marked increase in risk for hospitalized patients, particularly those over the age of 40. This is in agreement with what previous research had shown, particularly regarding non-comorbid patients [37].

**Fig 7:**
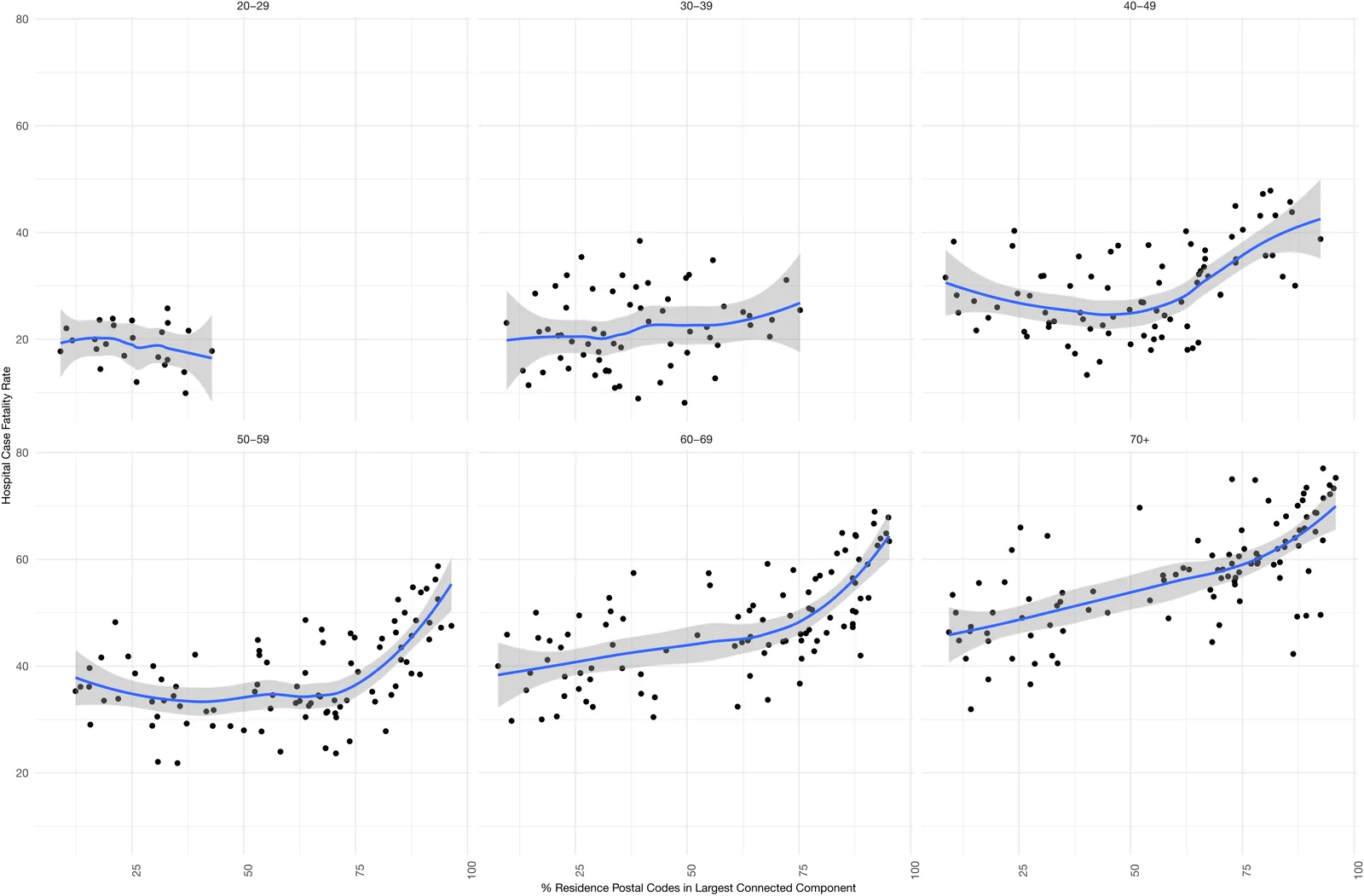
Association between hospital case fatality rate and the relative size of the largest connected component (LCC) in the hospital demand network. Each point represents an observation at a given time period. The LCC size is expressed as the percentage of nodes included in the component. Blue lines show LOESS regression fits. Panels correspond to different age groups.

This phase transition in risk suggests that the LCC, as a global measure of network strain, may be capturing the cumulative effects of increased pressure on individual hospitals. During periods of high demand, hospitals experience a higher patient load, potentially leading to resource shortages, longer wait times, and diminished quality of care: all of which can contribute to worse outcomes for patients, as reported in comparisons of temporary ICUs units with conventional ICUs [38] and in studies of poor ICU bed availability [39]. The non-linear nature of this relationship underscores the complexity of the healthcare system, where local interactions and constraints can lead to emergent global phenomena that significantly impact patient care.

Understanding this dynamic is crucial for managing hospital resources and mitigating risk during periods of high demand. It highlights the need for interventions that not only address hospital capacity at a local level but also consider the broader network effects that can exacerbate strain across the healthcare system. Recognizing and responding to these critical phase transitions could be key to improving patient outcomes in future public health emergencies.

## Conclusions

The COVID-19 pandemic in Mexico City illustrates the complex and dynamic nature of public health crises, driven by multiple interacting layers: epidemic spread, social behavior, and systemic constraints. The crisis demanded a multifaceted response, and adaptive measures, such as temporary COVID-19 care units, proved effective in absorbing sudden surges in hospital demand. Facilities like the one established in the city’s northwest played a crucial role during peak periods and offer a valuable blueprint for future emergency deployments.

The usefulness of these interventions underscores the need to institutionalize rapid-response strategies that enhance system flexibility and resilience. Expanding the capacity to deploy temporary medical facilities, alongside more agile resource allocation models, can help mitigate future shocks to healthcare infrastructure.

Additionally, incorporating complexity science approaches, such as network analysis of hospital demand, offers powerful early warning tools. These methods can detect critical transitions and emerging strains, enabling timely, targeted responses. Embedding such tools into public health planning will be key to anticipating and managing the challenges of future health emergencies.
