# Supplementary material for: Complexity of hospital demand during the COVID-19 pandemic in Mexico City
Source: PLoS One. 2026 Sep 30;21(9):e0358798. doi: 10.1371/journal.pone.0358798 (PMC13626320; doi:10.1371/journal.pone.0358798)
Supplement: Supplementary file 1 [file pone.0358798.s001.docx]

**Response to reviewer:**

**First, the manuscript should be reorganized to fully comply with the journal’s submission guidelines. In particular, the structure of the manuscript should follow the standard format required by PLOS ONE, as indicated in the submission instructions.**

We thank the reviewer for their helpful comments. We have reorganized the manuscript to follow the standard format used in PLOS ONE. We decided on keeping the section of “results and discussion” in its combined form, as we find that it makes more sense for the narrative flow. Considering that other manuscripts in PLOS ONE do have such combined results and discussion sections, we believe that this does fit the format of the journal.

**Second, in our opinion the Introduction requires further development. Given the large body of international literature on COVID-19 hospital demand, health-system stress, and network-based approaches, the state of the art should be strengthened. We encourage the authors to better contextualize their work within recent international studies and to clearly articulate the specific novelty and contribution of the present study relative to existing publications.**

We thank the reviewer for this comment. In response, we have strengthened the Introduction by incorporating additional international literature on COVID-19 hospital demand, health-system stress, and network-based approaches, and by clarifying the specific contribution of our work.

First, to contextualize our study within the broader international literature on health-system stress and disruption during COVID-19, we incorporated large-scale comparative and systems-level analyses (References 1-4), as well as studies addressing spatial and temporal dynamics of the pandemic (References 5-7) and hospital surge capacity (Ref. 8). These additions situate our work within established efforts to understand healthcare system strain under pandemic conditions.

Second, we expanded the discussion of health systems as complex adaptive systems by including references that explicitly frame healthcare under stress in terms of non-linear dynamics and phase transitions (References 11, 13, 14). This provides the conceptual basis for interpreting systemic responses to demand shocks.

Third, we strengthened the network-science component of the Introduction by incorporating foundational literature (Reference 16) and prior applications of network approaches to epidemic processes (References. 17-20), as well as recent studies specifically modeling healthcare systems and patient flows as networks (References. 21-24). These references position our work within existing network-based analyses of healthcare demand and system organization.

Finally, we clarified the novelty and contribution of the present study by explicitly framing hospital demand as a time-resolved bipartite network linking residential areas and healthcare facilities, and by focusing on the emergence of large connected components as indicators of system-wide reorganization under stress. Unlike prior studies, which have primarily examined spatial patterns, mobility, or patient flows in isolation, our approach integrates these elements into a unified network framework and directly relates changes in this system representation to patient outcomes.

**The methodological description should be clarified, particularly regarding the construction of the bipartite network and the definition and interpretation of the largest connected component as an indicator of system strain.**

We have modified the methods section to formally define both the bipartite network model used in this work, as well as the definition of largest and giant connected components for a general audience. We have clarified in this section that strain is referring to hospitalization demand. The larger interpretation of the relationship between network structure and this hospital demand, as well as the impact on outcomes, is covered in the results and discussion section.

**The manuscript would benefit from language editing and stylistic improvements, including reducing the excessive use of em-dashes (—) within sentences, which in several cases interrupts the flow of the text (e.g., “Adaptive measures—such as the deployment of temporary COVID-19 units—proved effective…”).**

We have modified the general writing of the manuscript to simplify the reading flow. We have reduced the use of em-dashes, as well as avoided the overuse of parenthetical phrases.

**Please ensure that all figures are fully interpretable as standalone elements, with clearer captions explaining variables, temporal coverage, and analytical assumptions.**

We have modified all captions to be self-contained and describe all variables needed for their interpretation.

**We note that the grant information you provided in the ‘Funding Information’ and ‘Financial Disclosure’ sections do not match. When you resubmit, please ensure that you provide the correct grant numbers for the awards you received for your study in the ‘Funding Information’ section.**

Done.

**Please state what role the funders took in the study. If the funders had no role, please state: "The funders had no role in study design, data collection and analysis, decision to publish, or preparation of the manuscript."**

Done.

**We note that you have provided funding information that is currently declared in your Funding Statement. However, funding information should not appear in the Acknowledgments section or other areas of your manuscript. Please remove any funding-related text from the manuscript, and ensure that this information is included only in the Funding Statement section of the submission form.**

Done.

**We note that Figures 1 and 2 in your submission contain map/satellite images which may be copyrighted. In order for us to publish this paper, we need to ensure that these images are not subject to copyright restrictions. We cannot publish previously copyrighted maps or satellite images created using proprietary data, such as Google software, or copied from other publications. Please ensure that the maps used in your figures are either original, based on open data, or appropriately licensed, and provide the source of the map data in the figure captions.**

These maps were generated by ourselves using open data and open source software. The geometries (shapefiles) for map generation were obtained from the mexican National Institute of Statistics and Geography (INEGI), using a cartography that is free and open. The actual visualizations were generated by ourselves using Rspatial packages. We have included the information of the INEGI shapefiles in the manuscripts to avoid giving the impression that maps were generated with proprietary data.

We thank the reviewer for their comments and suggestions, which surely improve the quality of our manuscript.

**Please include the following items when submitting your revised manuscript:
 A letter that responds to each point raised by the editor and reviewer(s).
 A marked-up copy of your manuscript that highlights changes made to the original version.
 An unmarked version of your revised manuscript.**

We have included the requested items.
