# Supplementary material for: Complexity of hospital demand during the COVID-19 pandemic in Mexico City
Source: PLoS One. 2026 Sep 30;21(9):e0358798. doi: 10.1371/journal.pone.0358798 (PMC13626320; doi:10.1371/journal.pone.0358798)
Supplement: Supplementary file 2 [file pone.0358798.s002.docx]

Response to reviewers:

**Reviewer comment: “p12 (Introduction) he scale of this ... => The scale of this”**

Response: All located typos such as this have been addressed.

**Reviewer comment: “According to the formulas ‘largest connected component (LCC)’ and ‘giant component’ are identical, is this correct?”**

Response: We have modified the Methods section to address this question. The largest connected component is now correctly defined as the connected component containing the greatest number of nodes. A giant component is defined as the LCC only when its relative size exceeds one half of the nodes in the network. Thus, every weekly network has an LCC, but only some weeks exhibit a giant component.

**Reviewer comment: “All network analyses were performed using {igraph} version 2.2 (25) for R (version 4.5). => Igraph”**

Response: We have reformatted the reference as suggested.

**Reviewer comment: “p20 ICU´s => ICUs”**

Response: Corrected throughout the manuscript. We also replaced the Spanish acronym “UCI” with “ICU/ICUs” where appropriate.

**Reviewer comment: “Please insert page numbers.”**

Response: Page numbers have been inserted in the footer of the revised manuscript.

**Reviewer comment: “There are still a few minor spelling and grammar errors... read through the manuscript carefully one more time.”**

Response: We performed an additional proofreading pass and corrected minor spelling, punctuation, spacing, and grammar issues in the Introduction, Methods, Results and Discussion, and Conclusions sections.
