# Supplementary material for: Complexity of hospital demand during the COVID-19 pandemic in Mexico City
Source: PLoS One. 2026 Sep 30;21(9):e0358798. doi: 10.1371/journal.pone.0358798 (PMC13626320; doi:10.1371/journal.pone.0358798)
Supplement: Peer review history

# Editor report: Decision Letter 0

**DOI:** 10.1371/journal.pone.0358798.r001

**Authors:** Benedetto Schiavo

16 Mar 2026

PLOS One

Dear Dr. de Anda-Jáuregui,

Thank you for submitting your manuscript to PLOS ONE. After careful consideration, we feel that it has merit but does not fully meet PLOS ONE’s publication criteria as it currently stands. Therefore, we invite you to submit a revised version of the manuscript that addresses the points raised during the review process.

The topic addressed in your study is relevant and potentially of interest to the readership. However, before the manuscript can be sent for external peer review, several issues need to be addressed.

First, the manuscript should be reorganized to fully comply with the journal’s submission guidelines (https://journals.plos.org/plosone/s/submission-guidelines#loc-manuscript-organization). In particular, the structure of the manuscript should follow the standard format required by PLOS ONE, as indicated in the submission instructions.

Second, in our opinion the Introduction requires further development. Given the large body of international literature on COVID-19 hospital demand, health-system stress, and network-based approaches, the state of the art should be strengthened. We encourage the authors to better contextualize their work within recent international studies and to clearly articulate the specific novelty and contribution of the present study relative to existing publications.

In addition, please consider addressing the following points before resubmission:

- The methodological description should be clarified, particularly regarding the construction of the bipartite network and the definition and interpretation of the largest connected component as an indicator of system strain.
- The manuscript would benefit from language editing and stylistic improvements, including reducing the excessive use of em-dashes (—) within sentences, which in several cases interrupts the flow of the text (e.g., “Adaptive measures—such as the deployment of temporary COVID-19 units—proved effective…”).
- Please ensure that all figures are fully interpretable as standalone elements, with clearer captions explaining variables, temporal coverage, and analytical assumptions.

Once these issues are satisfactorily resolved, the manuscript will be reconsidered for peer review.

Please submit your revised manuscript by Apr 30 2026 11:59PM. If you will need more time than this to complete your revisions, please reply to this message or contact the journal office at plosone@plos.org. When you're ready to submit your revision, log on to https://www.editorialmanager.com/pone/ and select the 'Submissions Needing Revision' folder to locate your manuscript file.

Please include the following items when submitting your revised manuscript:

- A letter that responds to each point raised by the academic editor and reviewer(s). You should upload this letter as a separate file labeled 'Response to Reviewers'.
- A marked-up copy of your manuscript that highlights changes made to the original version. You should upload this as a separate file labeled 'Revised Manuscript with Track Changes'.
- An unmarked version of your revised paper without tracked changes. You should upload this as a separate file labeled 'Manuscript'.

If you would like to make changes to your financial disclosure, please include your updated statement in your cover letter. Guidelines for resubmitting your figure files are available below the reviewer comments at the end of this letter.

If applicable, we recommend that you deposit your laboratory protocols in protocols.io to enhance the reproducibility of your results. Protocols.io assigns your protocol its own identifier (DOI) so that it can be cited independently in the future. For instructions see: https://journals.plos.org/plosone/s/submission-guidelines#loc-laboratory-protocols. Additionally, PLOS ONE offers an option for publishing peer-reviewed Lab Protocol articles, which describe protocols hosted on protocols.io. Read more information on sharing protocols at https://plos.org/protocols?utm_medium=editorial-email&utm_source=authorletters&utm_campaign=protocols.

We look forward to receiving your revised manuscript.

Kind regards,

Benedetto Schiavo, Ph.D.

Academic Editor

PLOS One

Journal Requirements:

When submitting your revision, we need you to address these additional requirements.

1. Please ensure that your manuscript meets PLOS ONE's style requirements, including those for file naming. The PLOS ONE style templates can be found at

https://journals.plos.org/plosone/s/file?id=wjVg/PLOSOne_formatting_sample_main_body.pdf and

https://journals.plos.org/plosone/s/file?id=ba62/PLOSOne_formatting_sample_title_authors_affiliations.pdf

2. We note that the grant information you provided in the ‘Funding Information’ and ‘Financial Disclosure’ sections do not match.

When you resubmit, please ensure that you provide the correct grant numbers for the awards you received for your study in the ‘Funding Information’ section.

3. Thank you for stating the following financial disclosure:

“CONACYT Grant to Guillermo de Anda Jáuregui”

Please state what role the funders took in the study. If the funders had no role, please state: "The funders had no role in study design, data collection and analysis, decision to publish, or preparation of the manuscript."

If this statement is not correct you must amend it as needed.

Please include this amended Role of Funder statement in your cover letter; we will change the online submission form on your behalf.

4. Thank you for stating the following in the Acknowledgments Section of your manuscript:

“The authors thank the Mexico City Ministry of Health and the National Epidemiological Surveillance System (SISVER) for data access and logistical support. This work was partially supported by CONACYT project 320557 (to GA-J).”

We note that you have provided funding information that is currently declared in your Funding Statement. However, funding information should not appear in the Acknowledgments section or other areas of your manuscript. We will only publish funding information present in the Funding Statement section of the online submission form.

Please remove any funding-related text from the manuscript and let us know how you would like to update your Funding Statement. Currently, your Funding Statement reads as follows:

“CONACYT Grant to Guillermo de Anda Jáuregui”

Please include your amended statements within your cover letter; we will change the online submission form on your behalf.

5.  We note that Figures 1 and 2 in your submission contain map/satellite images which may be copyrighted. All PLOS content is published under the Creative Commons Attribution License (CC BY 4.0), which means that the manuscript, images, and Supporting Information files will be freely available online, and any third party is permitted to access, download, copy, distribute, and use these materials in any way, even commercially, with proper attribution. For these reasons, we cannot publish previously copyrighted maps or satellite images created using proprietary data, such as Google software (Google Maps, Street View, and Earth). For more information, see our copyright guidelines: http://journals.plos.org/plosone/s/licenses-and-copyright.

We require you to either (1) present written permission from the copyright holder to publish these figures specifically under the CC BY 4.0 license, or (2) remove the figures from your submission:

a. You may seek permission from the original copyright holder of Figures 1 and 2 to publish the content specifically under the CC BY 4.0 license.

We recommend that you contact the original copyright holder with the Content Permission Form (http://journals.plos.org/plosone/s/file?id=7c09/content-permission-form.pdf) and the following text:

“I request permission for the open-access journal PLOS ONE to publish XXX under the Creative Commons Attribution License (CCAL) CC BY 4.0 (http://creativecommons.org/licenses/by/4.0/). Please be aware that this license allows unrestricted use and distribution, even commercially, by third parties. Please reply and provide explicit written permission to publish XXX under a CC BY license and complete the attached form.”

Please upload the completed Content Permission Form or other proof of granted permissions as an "Other" file with your submission.

In the figure caption of the copyrighted figure, please include the following text: “Reprinted from [ref] under a CC BY license, with permission from [name of publisher], original copyright [original copyright year].”

b. If you are unable to obtain permission from the original copyright holder to publish these figures under the CC BY 4.0 license or if the copyright holder’s requirements are incompatible with the CC BY 4.0 license, please either i) remove the figure or ii) supply a replacement figure that complies with the CC BY 4.0 license. Please check copyright information on all replacement figures and update the figure caption with source information. If applicable, please specify in the figure caption text when a figure is similar but not identical to the original image and is therefore for illustrative purposes only.

The following resources for replacing copyrighted map figures may be helpful:

USGS National Map Viewer (public domain): http://viewer.nationalmap.gov/viewer/

The Gateway to Astronaut Photography of Earth (public domain): http://eol.jsc.nasa.gov/sseop/clickmap/

Maps at the CIA (public domain): https://www.cia.gov/library/publications/the-world-factbook/index.html and https://www.cia.gov/library/publications/cia-maps-publications/index.html

NASA Earth Observatory (public domain): http://earthobservatory.nasa.gov/

Landsat: http://landsat.visibleearth.nasa.gov/

USGS EROS (Earth Resources Observatory and Science (EROS) Center) (public domain): http://eros.usgs.gov/#

Natural Earth (public domain): http://www.naturalearthdata.com/

To ensure your figures meet our technical requirements, please review our figure guidelines: https://journals.plos.org/plosone/s/figures

You may also use PLOS’s free figure tool, NAAS, to help you prepare publication quality figures: https://journals.plos.org/plosone/s/figures#loc-tools-for-figure-preparation.

NAAS will assess whether your figures meet our technical requirements by comparing each figure against our figure specifications.

# Author comment: Author response to Decision Letter 1

**DOI:** 10.1371/journal.pone.0358798.r002

22 Apr 2026

Response to reviewer:

First, the manuscript should be reorganized to fully comply with the journal’s submission guidelines. In particular, the structure of the manuscript should follow the standard format required by PLOS ONE, as indicated in the submission instructions.

We thank the reviewer for their helpful comments. We have reorganized the manuscript to follow the standard format used in PLOS ONE. We decided on keeping the section of “results and discussion” in its combined form, as we find that it makes more sense for the narrative flow. Considering that other manuscripts in PLOS ONE do have such combined results and discussion sections, we believe that this does fit the format of the journal.

Second, in our opinion the Introduction requires further development. Given the large body of international literature on COVID-19 hospital demand, health-system stress, and network-based approaches, the state of the art should be strengthened. We encourage the authors to better contextualize their work within recent international studies and to clearly articulate the specific novelty and contribution of the present study relative to existing publications.

We thank the reviewer for this comment. In response, we have strengthened the Introduction by incorporating additional international literature on COVID-19 hospital demand, health-system stress, and network-based approaches, and by clarifying the specific contribution of our work.

First, to contextualize our study within the broader international literature on health-system stress and disruption during COVID-19, we incorporated large-scale comparative and systems-level analyses (References 1-4), as well as studies addressing spatial and temporal dynamics of the pandemic (References 5-7) and hospital surge capacity (Ref. 8). These additions situate our work within established efforts to understand healthcare system strain under pandemic conditions.

Second, we expanded the discussion of health systems as complex adaptive systems by including references that explicitly frame healthcare under stress in terms of non-linear dynamics and phase transitions (References 11, 13, 14). This provides the conceptual basis for interpreting systemic responses to demand shocks.

Third, we strengthened the network-science component of the Introduction by incorporating foundational literature (Reference 16) and prior applications of network approaches to epidemic processes (References. 17-20), as well as recent studies specifically modeling healthcare systems and patient flows as networks (References. 21-24). These references position our work within existing network-based analyses of healthcare demand and system organization.

Finally, we clarified the novelty and contribution of the present study by explicitly framing hospital demand as a time-resolved bipartite network linking residential areas and healthcare facilities, and by focusing on the emergence of large connected components as indicators of system-wide reorganization under stress. Unlike prior studies, which have primarily examined spatial patterns, mobility, or patient flows in isolation, our approach integrates these elements into a unified network framework and directly relates changes in this system representation to patient outcomes.

The methodological description should be clarified, particularly regarding the construction of the bipartite network and the definition and interpretation of the largest connected component as an indicator of system strain.

We have modified the methods section to formally define both the bipartite network model used in this work, as well as the definition of largest and giant connected components for a general audience. We have clarified in this section that strain is referring to hospitalization demand. The larger interpretation of the relationship between network structure and this hospital demand, as well as the impact on outcomes, is covered in the results and discussion section.

The manuscript would benefit from language editing and stylistic improvements, including reducing the excessive use of em-dashes (—) within sentences, which in several cases interrupts the flow of the text (e.g., “Adaptive measures—such as the deployment of temporary COVID-19 units—proved effective…”).

We have modified the general writing of the manuscript to simplify the reading flow. We have reduced the use of em-dashes, as well as avoided the overuse of parenthetical phrases.

Please ensure that all figures are fully interpretable as standalone elements, with clearer captions explaining variables, temporal coverage, and analytical assumptions.

We have modified all captions to be self-contained and describe all variables needed for their interpretation.

We note that the grant information you provided in the ‘Funding Information’ and ‘Financial Disclosure’ sections do not match. When you resubmit, please ensure that you provide the correct grant numbers for the awards you received for your study in the ‘Funding Information’ section.

Done.

Please state what role the funders took in the study. If the funders had no role, please state: "The funders had no role in study design, data collection and analysis, decision to publish, or preparation of the manuscript."

Done.

We note that you have provided funding information that is currently declared in your Funding Statement. However, funding information should not appear in the Acknowledgments section or other areas of your manuscript. Please remove any funding-related text from the manuscript, and ensure that this information is included only in the Funding Statement section of the submission form.

Done.

We note that Figures 1 and 2 in your submission contain map/satellite images which may be copyrighted. In order for us to publish this paper, we need to ensure that these images are not subject to copyright restrictions. We cannot publish previously copyrighted maps or satellite images created using proprietary data, such as Google software, or copied from other publications. Please ensure that the maps used in your figures are either original, based on open data, or appropriately licensed, and provide the source of the map data in the figure captions.

These maps were generated by ourselves using open data and open source software. The geometries (shapefiles) for map generation were obtained from the mexican National Institute of Statistics and Geography (INEGI), using a cartography that is free and open. The actual visualizations were generated by ourselves using Rspatial packages. We have included the information of the INEGI shapefiles in the manuscripts to avoid giving the impression that maps were generated with proprietary data.

We thank the reviewer for their comments and suggestions, which surely improve the quality of our manuscript.

Please include the following items when submitting your revised manuscript:

A letter that responds to each point raised by the editor and reviewer(s).

A marked-up copy of your manuscript that highlights changes made to the original version.

An unmarked version of your revised manuscript.

We have included the requested items.

Attachment Submitted filename: Response to reviewer - PLOS complexity hospitalization.docx

# Peer review: Decision Letter 1

**DOI:** 10.1371/journal.pone.0358798.r003

**Authors:** Benedetto Schiavo

1 Jun 2026

Dear Dr. de Anda-Jáuregui,

Thank you for submitting your manuscript to PLOS ONE. After careful consideration, we feel that it has merit but does not fully meet PLOS ONE’s publication criteria as it currently stands. Therefore, we invite you to submit a revised version of the manuscript that addresses the points raised during the review process.

The manuscript has improved substantially and, in my opinion, is now suitable for publication. Before final acceptance, I kindly ask the authors to address the minor issues listed below, carefully proofread the manuscript for any remaining typographical or grammatical errors, and ensure that page numbers are included in the revised version.

Please submit your revised manuscript by Jul 16 2026 11:59PM. If you will need more time than this to complete your revisions, please reply to this message or contact the journal office at plosone@plos.org. When you're ready to submit your revision, log on to https://www.editorialmanager.com/pone/ and select the 'Submissions Needing Revision' folder to locate your manuscript file.

- A letter that responds to each point raised by the academic editor and reviewer(s). You should upload this letter as a separate file labeled 'Response to Reviewers'.
- A marked-up copy of your manuscript that highlights changes made to the original version. You should upload this as a separate file labeled 'Revised Manuscript with Track Changes'.
- An unmarked version of your revised paper without tracked changes. You should upload this as a separate file labeled 'Manuscript'.

If you would like to make changes to your financial disclosure, please include your updated statement in your cover letter. Guidelines for resubmitting your figure files are available below the reviewer comments at the end of this letter.

If applicable, we recommend that you deposit your laboratory protocols in protocols.io to enhance the reproducibility of your results. Protocols.io assigns your protocol its own identifier (DOI) so that it can be cited independently in the future. For instructions see: https://journals.plos.org/plosone/s/submission-guidelines#loc-laboratory-protocols. Additionally, PLOS ONE offers an option for publishing peer-reviewed Lab Protocol articles, which describe protocols hosted on protocols.io. Read more information on sharing protocols at https://plos.org/protocols?utm_medium=editorial-email&utm_source=authorletters&utm_campaign=protocols.

As the corresponding author, your ORCID iD is verified in the submission system and will appear in the published article. PLOS supports the use of ORCID, and we encourage all coauthors to register for an ORCID iD and use it as well. Please encourage your coauthors to verify their ORCID iD within the submission system before final acceptance, as unverified ORCID iDs will not appear in the published article. *Only*  the individual author can complete the verification step; PLOS staff *cannot*  verify ORCID iDs on behalf of authors.

We look forward to receiving your revised manuscript.

Kind regards,

Benedetto Schiavo, Ph.D.

Academic Editor

PLOS One

Journal Requirements:

If the reviewer comments include a recommendation to cite specific previously published works, please review and evaluate these publications to determine whether they are relevant and should be cited. There is no requirement to cite these works unless the editor has indicated otherwise.

Please review your reference list to ensure that it is complete and correct. If you have cited papers that have been retracted, please include the rationale for doing so in the manuscript text, or remove these references and replace them with relevant current references. Any changes to the reference list should be mentioned in the rebuttal letter that accompanies your revised manuscript. If you need to cite a retracted article, indicate the article’s retracted status in the References list and also include a citation and full reference for the retraction notice.

[Note: HTML markup is below. Please do not edit.]

Reviewers' comments:

Reviewer's Responses to Questions

**Comments to the Author**

Reviewer #1: All comments have been addressed

**********

2. Is the manuscript technically sound, and do the data support the conclusions?

Reviewer #1: (No Response)

**********

3. Has the statistical analysis been performed appropriately and rigorously?

Reviewer #1: (No Response)

**********

4. Have the authors made all data underlying the findings in their manuscript fully available?

The PLOS Data policy

Reviewer #1: (No Response)

**********

5. Is the manuscript presented in an intelligible fashion and written in standard English?

Reviewer #1: (No Response)

**********

Reviewer #1: Dear authors,

according to my assessment you have addressed all comments raised in the previous review round and have documented your revisions in detail using track changes. I agree that the “Results and Discussion” sections can be combined into a single chapter.

There are still a few minor spelling and grammar errors (few examples below), so I recommend reading through the manuscript carefully one more time.

p12 (Introduction)

he scale of this ... => The scale of this

p 15

According to the formulas 'largest connected component (LCC)' and 'giant component' are identical, is this correct?

All network analyses were performed using {igraph} version 2.2 (25) for R (version 4.5). => Igraph

p20

ICU´s => ICUs

Please insert page numbers.

**********

what does this mean?). If published, this will include your full peer review and any attached files.

If you choose “no”, your identity will remain anonymous but your review may still be made public.

**Do you want your identity to be public for this peer review?**  For information about this choice, including consent withdrawal, please see our Privacy Policy

Reviewer #1: No

**********

[NOTE: If reviewer comments were submitted as an attachment file, they will be attached to this email and accessible via the submission site. Please log into your account, locate the manuscript record, and check for the action link "View Attachments". If this link does not appear, there are no attachment files.]

To ensure your figures meet our technical requirements, please review our figure guidelines: https://journals.plos.org/plosone/s/figures

You may also use PLOS’s free figure tool, NAAS, to help you prepare publication quality figures: https://journals.plos.org/plosone/s/figures#loc-tools-for-figure-preparation.

NAAS will assess whether your figures meet our technical requirements by comparing each figure against our figure specifications.

# Author comment: Author response to Decision Letter 2

**DOI:** 10.1371/journal.pone.0358798.r004

3 Sep 2026

Response to reviewers:

Reviewer comment: “p12 (Introduction) he scale of this ... => The scale of this”

Response: All located typos such as this have been addressed.

Reviewer comment: “According to the formulas ‘largest connected component (LCC)’ and ‘giant component’ are identical, is this correct?”

Response: We have modified the Methods section to address this question. The largest connected component is now correctly defined as the connected component containing the greatest number of nodes. A giant component is defined as the LCC only when its relative size exceeds one half of the nodes in the network. Thus, every weekly network has an LCC, but only some weeks exhibit a giant component.

Reviewer comment: “All network analyses were performed using {igraph} version 2.2 (25) for R (version 4.5). => Igraph”

Response: We have reformatted the reference as suggested.

Reviewer comment: “p20 ICU´s => ICUs”

Response: Corrected throughout the manuscript. We also replaced the Spanish acronym “UCI” with “ICU/ICUs” where appropriate.

Reviewer comment: “Please insert page numbers.”

Response: Page numbers have been inserted in the footer of the revised manuscript.

Reviewer comment: “There are still a few minor spelling and grammar errors... read through the manuscript carefully one more time.”

Response: We performed an additional proofreading pass and corrected minor spelling, punctuation, spacing, and grammar issues in the Introduction, Methods, Results and Discussion, and Conclusions sections.

Attachment Submitted filename: Response_to_Reviewers_PONE-D-26-08590R1_m.docx

# Editor report: Decision Letter 2

**DOI:** 10.1371/journal.pone.0358798.r005

**Authors:** Benedetto Schiavo

6 Sep 2026

Complexity of hospital demand during the COVID-19 pandemic in Mexico City

PONE-D-26-08590R2

Dear Dr. de Anda-Jáuregui,

We’re pleased to inform you that your manuscript has been judged scientifically suitable for publication and will be formally accepted for publication once it meets all outstanding technical requirements.

Within one week, you’ll receive an e-mail detailing the required amendments. When these have been addressed, you’ll receive a formal acceptance letter and your manuscript will be scheduled for publication.

An invoice will be generated when your article is formally accepted. Please note, if your institution has a publishing partnership with PLOS and your article meets the relevant criteria, all or part of your publication costs will be covered. Please make sure your user information is up-to-date by logging into Editorial Manager at Editorial Manager® and clicking the ‘Update My Information' link at the top of the page. For questions related to billing, please contact billing support.

If your institution or institutions have a press office, please notify them about your upcoming paper to help maximize its impact. If they’ll be preparing press materials, please inform our press team as soon as possible -- no later than 48 hours after receiving the formal acceptance. Your manuscript will remain under strict press embargo until 2 pm Eastern Time on the date of publication. For more information, please contact onepress@plos.org.

Kind regards,

Benedetto Schiavo, Ph.D.

Academic Editor

PLOS One

Additional Editor Comments (optional):

Reviewers' comments:

# Editor report: Acceptance letter

**DOI:** 10.1371/journal.pone.0358798.r006

**Authors:** Benedetto Schiavo

PONE-D-26-08590R2

PLOS One

Dear Dr. de Anda-Jáuregui,

I'm pleased to inform you that your manuscript has been deemed suitable for publication in PLOS One. Congratulations! Your manuscript is now being handed over to our production team.

At this stage, our production department will prepare your paper for publication. This includes ensuring the following:

* All references, tables, and figures are properly cited

* All relevant supporting information is included in the manuscript submission,

* There are no issues that prevent the paper from being properly typeset

You will receive further instructions from the production team, including instructions on how to review your proof when it is ready. Please keep in mind that we are working through a large volume of accepted articles, so please give us a few days to review your paper and let you know the next and final steps.

Lastly, if your institution or institutions have a press office, please let them know about your upcoming paper now to help maximize its impact. If they'll be preparing press materials, please inform our press team within the next 48 hours. Your manuscript will remain under strict press embargo until 2 pm Eastern Time on the date of publication. For more information, please contact onepress@plos.org.

You will receive an invoice from PLOS for your publication fee after your manuscript has reached the completed accept phase. If you receive an email requesting payment before acceptance or for any other service, this may be a phishing scheme. Learn how to identify phishing emails and protect your accounts at https://explore.plos.org/phishing.

If we can help with anything else, please email us at customercare@plos.org.

Thank you for submitting your work to PLOS One and supporting open access.

Kind regards,

PLOS One Editorial Office Staff

on behalf of

Dr. Benedetto Schiavo

Academic Editor

PLOS One
